# Association between Body Roundness Index and diastolic function in middle-aged and older adults: a cross-sectional study

**DOI:** 10.3389/fcvm.2025.1659587

**Published:** 2026-01-12

**Authors:** Yazheng Xie, Jin Wang, Bing He, Jing Bai, Nan Wang, Dongliang Liu, Qichao Wang, Haoran Wang, Qiaotao Xie

**Affiliations:** 1 Cardiovascular Institute of Luohe, Luohe Central Hospital, Luohe Medical College, Luohe, China; 2 Department of Orthopedics, The Second Affiliated Hospital of Luohe Medical College, Luohe, China

**Keywords:** adiposity, Body Roundness Index, cardiovascular disease, diastolic dysfunction, echocardiography

## Abstract

**Background:**

Diastolic dysfunction (DD) represents an early indicator of cardiac impairment and is strongly linked to adverse cardiovascular outcomes. While obesity-related indices have been associated with DD, the role of Body Roundness Index (BRI)-a novel adiposity measure reflecting body shape-remains unclear. This study aimed to evaluate the association between BRI and diastolic function in a community-based cohort.

**Methods:**

This cross-sectional analysis included 1,466 participants from the Longitudinal Investigation of Osteoarthritis and Cardiovascular Health Status cohort. BRI was calculated using a validated formula, and DD was assessed via echocardiographic parameters. BRI's optimal cutoff was derived via 1000-iteration bootstrap ROC analysis. Multivariable regression models were used to evaluate associations, adjusting for age, sex, blood pressure, lipid profile, and cardiovascular comorbidities.

**Results:**

Higher BRI was significantly associated with increased odds of DD. In fully adjusted logistic regression, each 1-unit increase in BRI was associated with 14.4% higher odds of DD (OR = 1.144, 95% CI: 1.046–1.251, *P* = 0.003). High BRI (≥4.2) was linked to 41.7% higher odds of DD (OR = 1.417, 95% CI: 1.114–1.804, *P* = 0.004). Robust regression confirmed BRI was inversely associated with septal e’ (*β* = −0.164, *P* = 8.10 × 10^−4^) and lateral e' velocities (*β*=−0.167, *P* = 5.59 × 10^−3^), and marginally positively associated with E/e' ratio (*β*=0.121, *P* = 0.053). Restricted cubic spline models showed a nonlinear association between BRI and DD probability (*P* < 0.001 for overall association, *P* = 0.006 for linearity). Interaction analyses indicated BRI's effect on DD was not modulated by blood pressure and lipid profiles. Subgroup analyses indicated a consistent trend of association between BRI and DD.

**Conclusions:**

BRI is nonlinearly and independently associated with impaired diastolic function in middle-aged and older adults, with modest diagnostic performance. These findings provided evidence on the link between body shape metrics and DD.

## Introduction

Cardiovascular diseases (CVDs) remain a leading cause of morbidity and mortality worldwide (1), with diastolic dysfunction (DD) emerging as a critical early marker of cardiac impairment. Diastolic dysfunction is associated with an increased risk of heart failure, arrhythmias, and cardiovascular events, underscoring the need for early identification of modifiable risk factors (2–7). While body mass index (BMI) has long been used to characterize adiposity, it fails to capture body shape and regional fat distribution, which may better reflect cardiometabolic risk (8–10). The Body Roundness Index (BRI), a novel adiposity metric derived from waist circumference and height, offers a more nuanced assessment of body shape independent of BMI (11, 12). Unlike BMI, BRI emphasizes central adiposity, a known contributor to cardiac remodeling and dysfunction. Preclinical and clinical studies have suggested that abdominal obesity is linked to impaired diastolic function, but the specific role of BRI in this context remains underexplored (13). Understanding this relationship is crucial for developing targeted screening strategies, as BRI may serve as a simple, non-invasive marker to identify individuals at risk for DD in primary care settings (14).

This cross-sectional study aims to fill this gap by investigating the association between BRI and diastolic function in a community-based cohort of middle-aged and older adults. We hypothesize that BRI is independently associated with DD, even after adjusting for traditional cardiovascular risk factors. By leveraging echocardiographic measures of diastolic function (e.g., E/e’ ratio, septal and lateral e’ velocities), we seek to characterize the nature of this association and explore potential effect modification by blood pressure, lipid profiles, and demographic factors. These findings may inform the integration of body shape metrics into routine cardiac health assessments, enhancing early detection and prevention of CVDs.

## Methods

### Study population

This cross-sectional analysis utilized baseline data from the Longitudinal Investigation of Osteoarthritis and Cardiovascular Health Status cohort, a prospective observational study (15) conducted in Luohe City and its surrounding areas in China between November 2023 and November 2024. The parent cohort aimed to examine the association between osteoarthritis and major adverse cardiovascular events (MACE), adhering to the Strengthening the Reporting of Observational Studies in Epidemiology (STROBE) statement (16).

Eligible participants were middle-aged and older adults (35–75 years) who had resided locally for ≥5 years, were willing to undergo cardiovascular and joint imaging (ultrasound and digital radiography), and could commit to ≥5 years of follow-up. Exclusion criteria included acute cardiovascular conditions (e.g., myocardial infarction, cardiogenic shock), malignancy, life-threatening trauma, severe psychiatric/cognitive impairment, or chronic kidney disease (estimated glomerular filtration rate <30 mL/min).

Of the 1,762 individuals enrolled in the parent cohort, 1,466 were included in this analysis after excluding 292 without sufficient echocardiographic data and 4 with missing waist circumference or height (required for BRI calculation).

### Measurements

BRI was calculated using the formula: BRI = 364.2–365.5 * sqrt{1 – [WC/(2 * π * Height/2)]^2}, in which waist circumference was measured at the umbilical level, and height was recorded using a stadiometer.

Transthoracic echocardiography was performed by trained sonographers using a Philips Epic-7 system (Philips Healthcare, Amsterdam, Netherlands). Key diastolic function parameters were measured and defined in accordance with the 2025 American Society of Echocardiography (ASE) guidelines (17): Mitral annular e’ velocity was measured via tissue Doppler imaging (TDI) at the septal and lateral mitral annulus in the apical four-chamber view, with average e’ calculated as the mean of septal and lateral e’ velocities, and age-dependent cutoffs for impaired left ventricular (LV) relaxation applied-specifically, average e’ < 9 cm/s for 20–39 years, average e’ < 7 cm/s for 40–65 years, and average e’ < 6.5 cm/s for those >65 years; the E/e’ ratio was calculated as the ratio of transmitral early diastolic velocity (E, measured via pulsed-wave Doppler at the mitral leaflet tips) to average e’ velocity, with an average E/e’ > 14 defined as elevated LV filling pressure. Diastolic dysfunction was determined via two conditions: (1) reduced e' velocity; (2) additional markers of diastolic impairment were considered: mitral E/A ratio ≤ 0.8 or ≥ 2, mitral E/e' ratio > 14, and left atrial volume index (LAVI) > 34 mL/m^2^. Finally, diastolic dysfunction was diagnosed if either of the following criteria were satisfied: (1) reduced e' velocity plus at least 1 abnormal additional marker, or (2) preserved e' velocity plus at least 2 abnormal additional markers; all other cases were classified as normal diastolic function (17, 18). All the measurements of cardiac ultrasound followed clinical quality control standards.

Besides, each participant provided a comprehensive set of clinical data including age, gender, smoking status (classified as current smoker or not), alcohol use (classified as yes or no), self-reported medical history data on the presence of hypertension, diabetes, coronary heart disease (CHD) and stroke which were confirmed by medical records when possible. Blood pressure measured in the sitting position with a calibrated sphygmomanometer where the average of two consecutive measurements was recorded, morning blood samples collected after at least 8 h of fasting to measure lipid profiles including total cholesterol (TC), low-density lipoprotein cholesterol (LDL-C), high-density lipoprotein cholesterol (HDL-C), triglycerides and blood glucose levels. BFR was calculated using a validated anthropometric formula: BFR = 1.20 × BMI + 0.23 × Age − 16.2 (5.4 for female).

### Statistical analysis

For baseline characteristics, continuous variables underwent normality testing using the Shapiro–Wilk test. Normally distributed variables were presented as mean ± standard deviation and compared via independent t-tests. Non-normally distributed variables were reported as median [interquartile range (IQR)] and compared using the Mann–Whitney U test. Categorical variables were expressed as frequencies (%) and analyzed via chi-square tests. To evaluate BRI's discriminatory ability for DD, a receiver operating characteristic (ROC) curve was constructed. A 1000-iteration bootstrap was performed to correct for optimism bias, generating the bootstrap-corrected area under the curve (AUC) and 95% confidence intervals (CIs). The optimal BRI cutoff was determined using the Youden index (sensitivity + specificity − 1), distinguishing participants into low BRI and high BRI groups. Additionally, the comparative discriminative performance of BRI vs. conventional adiposity indices was evaluated by constructing unadjusted ROC curves for waist circumference, body mass index (BMI), and body fat ratio (BFR), followed by comparison of their areas under the curve (AUCs). Logistic regression was used to assess the association between Body Roundness Index (BRI)-both as a continuous variable (per 1-unit increase) and a categorical variable (dichotomized at the optimal cutoff, rounded to 4.2 for clinical interpretability)-and DD, with three models constructed: Model 1 (unadjusted), Model 2 (adjusted for age and sex), and Model 3 [fully adjusted for age, sex, systolic blood pressure (SBP), diastolic blood pressure (DBP), TC, LDL-C, HDL-C, triglycerides, current smoking, alcohol use, hypertension, diabetes, CHD, and stroke], and odds ratios (ORs) with 95% confidence intervals (CIs) are reported. We also used regression models to evaluate the association between BRI and continuous diastolic function metrics (septal e’, lateral e’, E/e’ ratio). Prior to analysis, residual normality was assessed. If residuals were non-normal, robust regression with Huber-White standard errors was used to mitigate the impact of outliers; otherwise, the linear regression model was adopted. Models were adjusted similarly to logistic regression, and beta coefficients (*β*) with standard errors (SEs) were reported. A restricted cubic spline (RCS) model with 3 knots was used to assess the linearity of the association between BRI and DD probability, and *P-*values for overall association and nonlinearity are reported. Interaction effects between BRI and cardiovascular risk factors (blood pressure, lipid profiles) on DD were evaluated using multiplicative terms in regression models, with statistically significant interactions visualized. Subgroup analyses involved stratified analyses conducted by age (<50, 50–60, >60 years), sex, current smoking, alcohol use, hypertension, diabetes, CHD, and stroke, and forest plots were used to visualize ORs and 95% CIs for each subgroup. All analyses were performed using R software (version 4.4.2).

## Results

### Baseline characteristics by BRI category

**Figure 1:**
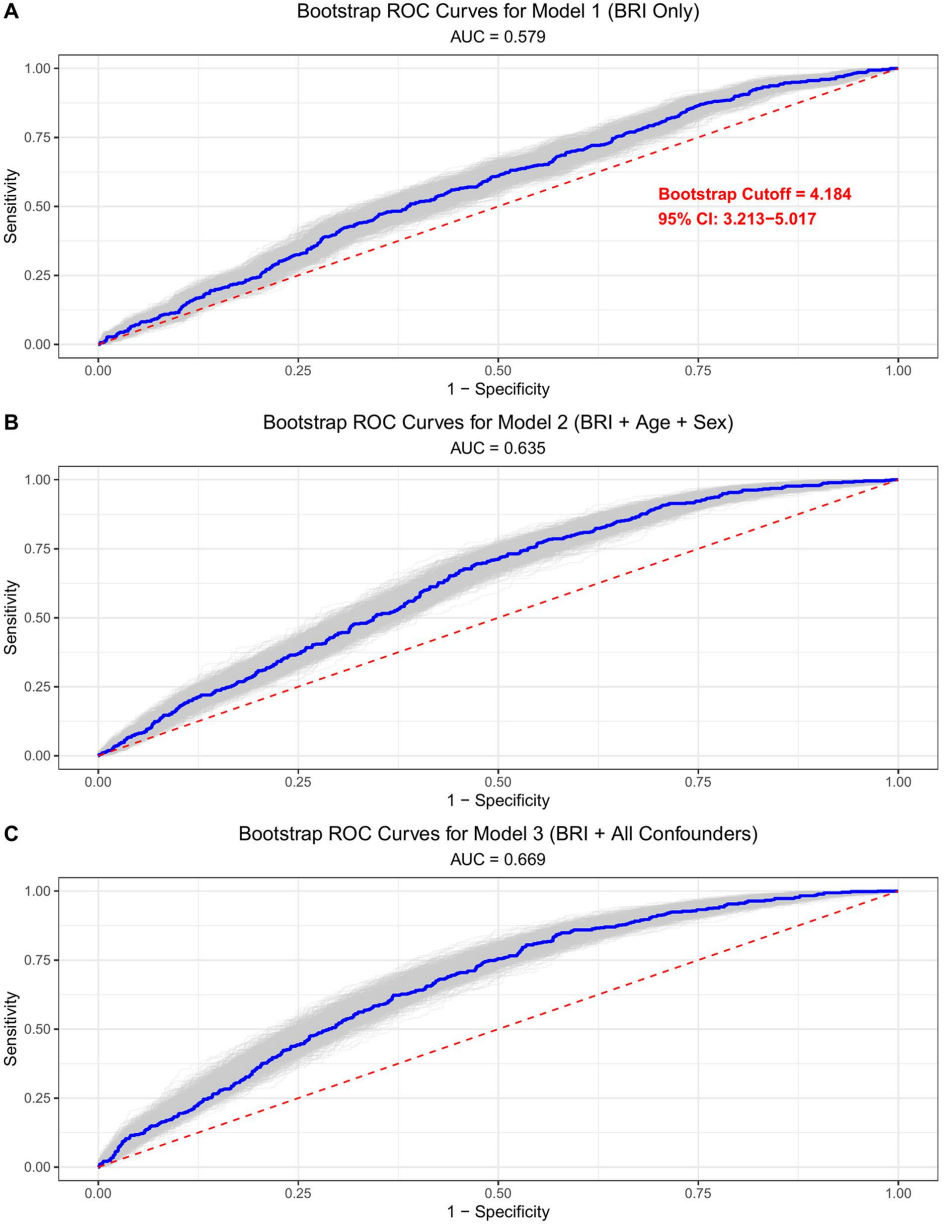
ROC curves illustrating the discriminative ability of BRI for identifying DD. Analyses were performed using 1000 bootstrap resamplings to assess stability; the optimal cutoff was determined by maximizing the Youden index (sensitivity + specificity − 1). **(A)** Model 1 is unadjusted. **(B)** Model 2 is adjusted for age and sex. **(C)** Model 3 is adjusted for all covariates (age, sex, systolic blood pressure, diastolic blood pressure, total cholesterol, low-density lipoprotein cholesterol, high-density lipoprotein cholesterol, triglycerides, current smoking, alcohol use, hypertension, diabetes, coronary heart disease, and stroke).

Using the optimal cutoff of 4.18 for BRI (derived from bootstrap ROC analysis, Figure 1A), participants were stratified into low BRI (<4.2, *n* = 662) and high BRI (≥4.2, *n* = 804) groups for clinical interpretability. Baseline characteristics differed significantly between groups (Table 1). High BRI participants had a higher median age (60 vs. 59 years, *P* = 0.040) and a higher prevalence of hypertension (45% vs. 33%, *P* < 0.001) and diabetes (10% vs. 6.8%, *P* = 0.022). Biochemical markers showed unfavorable profiles in the high BRI group, including lower high-density lipoprotein cholesterol (HDL-C: 1.35 vs. 1.43 mmol/L, *P* < 0.001) and higher triglycerides (1.80 vs. 1.39 mmol/L, *P* < 0.001), alongside higher systolic (152 vs. 150 mmHg, *P* < 0.001) and diastolic blood pressure (92 vs. 89 mmHg, *P* = 0.001). Anthropometrically, high BRI individuals had significantly larger waist circumference (94 vs. 80 cm, *P* < 0.001), higher body mass index (27.7 vs. 23.6 kg/m^2^, *P* < 0.001), and greater body fat ratio (0.38 vs. 0.27, *P* < 0.001). Echocardiographic parameters reflecting diastolic function also differed between groups. High BRI participants had lower septal e' velocity (6.70 vs. 7.22 cm/s, *P* < 0.001), lower lateral e’ velocity (9.2 vs. 9.8 cm/s, *P* < 0.001), and higher E/e' ratio (8.37 vs. 7.88, *P* = 0.009). The prevalence of DD, redefined per the 2025 American Society of Echocardiography (ASE) guidelines (either age-adjusted reduced average e' or elevated average E/e'), was significantly higher in the high BRI group (39% vs. 28%, *P* < 0.001). In contrast, left ventricular hypertrophy (6.5% vs. 5.9%, *P* = 0.735) and left atrial enlargement (21% vs. 19%, *P* = 0.501) showed no significant between-group differences.

**Table 1:** Baseline characteristics by BRI category (cutoff =4.2).

Variable	Overall *N* = 1,466^a^	Low BRI *N* = 662^a^	High BRI *N* = 804^a^	*P-*value^b^
Age (years)	59 (53, 67)	59 (52, 67)	60 (54, 68)	0.040
Male	600 (41%)	277 (42%)	323 (40%)	0.553
Current Smoking	321 (22%)	156 (24%)	165 (21%)	0.181
Alcohol use	135 (9.2%)	51 (8.2%)	84 (10%)	0.086
Hypertension	579 (39%)	220 (33%)	359 (45%)	<0.001
Diabetes	128 (8.7%)	45 (6.8%)	83 (10%)	0.022
Coronary Heart disease	96 (6.5%)	41 (6.2%)	55 (6.8%)	0.695
Stroke	164 (11%)	69 (10%)	95 (12%)	0.448
total cholesterol (mmol/L)	5.21 (4.22, 6.43) [*n* = 3]	5.13 (4.15, 6.46) [*n* = 1]	5.27 (4.28, 6.40) [*n* = 2]	0.377
Low-density lipoprotein cholesterol (mmol/L)	2.97 (2.19, 4.16) [*n* = 46]	2.95 (2.16, 4.21) [*n* = 25]	2.97 (2.23, 4.10) [*n* = 21]	0.869
High-density lipoprotein cholesterol (mmol/L)	1.39 (1.20, 1.61) [*n* = 4]	1.43 (1.23, 1.69) [*n* = 4]	1.35 (1.18, 1.57)	<0.001
Triglycerides (mmol/L)	1.64 (1.16, 2.30) [*n* = 1]	1.39 (1.05, 2.13) [*n* = 1]	1.80 (1.31, 2.44)	<0.001
Glucose (mmol/L)	5.60 (5.30, 6.30)	5.50 (5.20, 6.10)	5.70 (5.30, 6.50)	<0.001
Systolic blood pressure (mmHg)	151 (137, 163)	150 (134, 162)	152 (142, 163)	<0.001
Diastolic blood pressure (mmHg)	91 (81, 101)	89 (80, 100)	92 (82, 101)	0.001
Waist Circumference (cm)	88 (81, 95)	80 (76, 85)	94 (90, 99)	<0.001
Body mass index (kg/m^2^)	25.7 (23.6, 28.1)	23.6 (21.9, 25.0)	27.7 (26.0, 29.5)	<0.001
Body fat ratio	0.32 (0.24, 0.40)	0.27 (0.20, 0.33)	0.38 (0.28, 0.43)	<0.001
Left ventricular mass index (g/m^2^)	77 (67, 89) [*n* = 1]	77 (66, 89) [*n* = 1]	79 (67, 90)	0.034
Left ventricular ejection fraction (%)	68 (63, 72) [*n* = 4]	68 (64, 72) [*n* = 4]	68 (63, 71)	0.033
Left atrial volume index (mL/m^2^)	27 (21, 34) [*n* = 2]	27 (21, 34) [*n* = 1]	28 (22, 34) [*n* = 1]	0.002
Relative wall thickness	0.38 (0.34, 0.42) [*n* = 1]	0.37 (0.33, 0.41) [*n* = 1]	0.38 (0.34, 0.42)	<0.001
Mitral valve E/e’ (ratio)	8.12 (6.32, 10.16)	7.88 (6.24, 9.82)	8.37 (6.46, 10.43)	0.009
Mitral valve septal e’ velocity (cm/s)	6.94 (5.60, 8.89)	7.22 (5.83, 9.12)	6.70 (5.36, 8.62)	<0.001
Mitral valve lateral e’ velocity (cm/s)	9.5 (7.5, 11.6)	9.8 (7.9, 12.2)	9.2 (7.4, 11.2)	<0.001
Diastolic dysfunction	495 (34%)	185 (28%)	310 (39%)	<0.001
Left ventricular hypertrophy	91 (6.2%) [*n* = 1]	39 (5.9%) [*n* = 1]	52 (6.5%)	0.735
Left atrial enlargement	296 (20%) [*n* = 2]	128 (19%) [*n* = 1]	168 (21%) [*n* = 1]	0.501

a Median (IQR) [*n* = missing];

b Mann–Whitney U test; Pearson's Chi-squared test. [*n* = X] indicates the number of individuals lacking data of the measurement.

### BRI and diastolic dysfunction: logistic regression results

Bootstrap ROC analysis (1000 iterations) demonstrated BRI's discriminatory ability for DD, with a corrected area under the curve (AUC) of 0.67 (95% CI: 0.65–0.70, Figure 1). In head-to-head comparison using unadjusted ROC curves, BRI showed marginally higher AUC than waist circumference (0.579 vs. 0.570), BMI (0.555), and BFR (0.551) (Supplementary Figure S1). Multivariable logistic regression results for the association between BRI and DD are presented in Table 2. In the unadjusted model (Model 1), each 1-unit increase in BRI was associated with 23.2% higher odds of DD (OR = 1.249, 95% CI: 1.147–1.360, *P* = 5.6 × 10^−7^). After adjusting for age and sex (Model 2), the association remained significant (OR = 1.205, 95% CI: 1.108–1.31, *P* = 1.3 × 10^−5^). In the fully adjusted model (Model 3), which included cardiovascular risk factors (blood pressure, lipid profile, smoking, alcohol use) and comorbidities (hypertension, diabetes, coronary heart disease, stroke), the association was attenuated but remained statistically significant: each 1-unit BRI increase was associated with a 14.4% higher odds of DD (OR = 1.144, 95% CI: 1.046–1.251, *P* = 0.003). When comparing BRI categories, high BRI (≥4.2) was associated with 41.7% higher odds of DD in the fully adjusted model (OR = 1.417, 95% CI: 1.114–1.804, *P* = 0.004).

**Table 2:** Logistic regression assessing the association between BRI and DD.

BRI parameters	Model 1		Model 2		Model 3	
	OR (95% CI)	*P-*value	OR (95% CI)	*P-*value	OR (95% CI)	*P-*value
Each 1 unit increment	1.249 (1.147,1.360)	5.6E−07	1.205 (1.108,1.31)	1.3E−05	1.144 (1.046,1.251)	0.003
Low BRI (<4.2) vs. High BRI (≥4.2)	1.618 (1.297,2.019)	2.0E-05	1.573 (1.254,1.974)	9.1E-05	1.417 (1.114,1.804)	0.004

Model 1 is unadjusted. Model 2 is adjusted for age and sex. Model 3 is adjusted for all covariates (age, sex, systolic blood pressure, diastolic blood pressure, total cholesterol, low-density lipoprotein cholesterol, high-density lipoprotein cholesterol, triglycerides, current smoking, alcohol use, hypertension, diabetes, coronary heart disease, and stroke).

### BRI and diastolic function parameters: robust regression results

Robust regression analyses revealed inverse associations between BRI and diastolic function indices (Table 3). In Model 3, each 1-unit BRI increase was associated with a 0.164 cm/s decrease in septal e’ velocity (*β* = −0.164, SE = 0.049, *P* = 8.10 × 10^−4^) and a 0.167 cm/s decrease in lateral e’ velocity (*β* = −0.167, SE = 0.060, *P* = 5.59 × 10^−3^). The E/e’ ratio showed a positive association with BRI (*β* = 0.121, SE = 0.062, *P* = 0.053). These findings indicate that higher BRI is linked to reduced myocardial relaxation and increased filling pressure.

**Table 3:** Robust regression assessing the association between BRI and diastolic parameters.

Diastolic parameters	Model 1		Model 2		Model 3	
	*β* (SE)	*P-*value	*β* (SE)	*P-*value	*β* (SE)	*P-*value
Septal e'	−0.282 (0.048)	5.21E-09	-0.265 (0.048)	3.02E-08	−0.164 (0.049)	8.10E-04
Lateral e'	-0.319 (0.063)	4.95E-07	-0.283 (0.060)	2.40E-06	-0.167 (0.060)	5.59E-03
E/e'	0.253 (0.059)	1.91E-05	0.214 (0.060)	3.31E-04	0.121 (0.062)	0.053

Model 1 is unadjusted. Model 2 is adjusted for age and sex. Model 3 is adjusted for all covariates (age, sex, systolic blood pressure, diastolic blood pressure, total cholesterol, low-density lipoprotein cholesterol, high-density lipoprotein cholesterol, triglycerides, current smoking, alcohol use, hypertension, diabetes, coronary heart disease, and stroke).

### Nonlinearity and interaction effects

**Figure 2:**
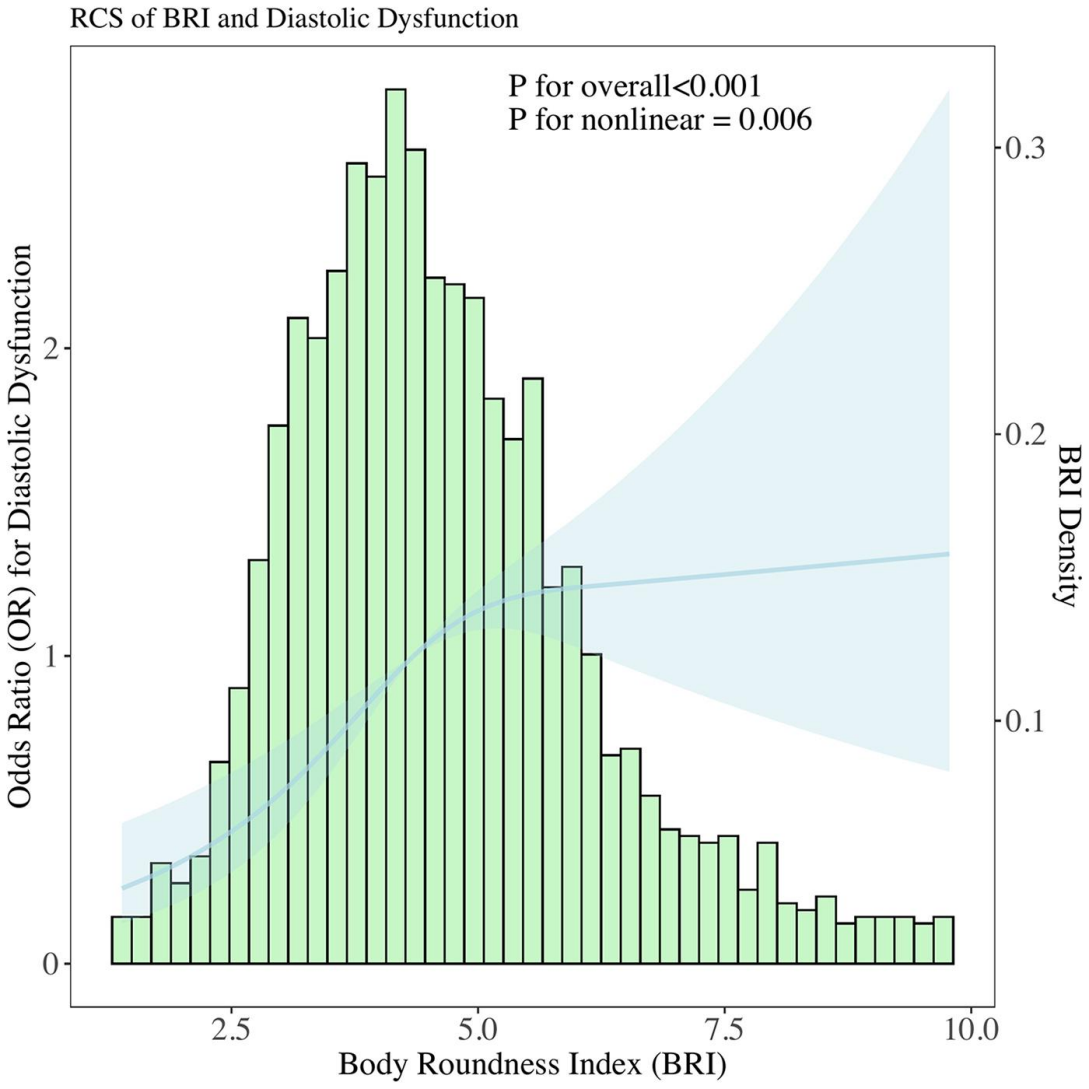
Histogram of BRI distribution overlaid with a restricted cubic spline model, examining the functional form of the association between BRI and the probability of DD. Spline included 3 knots to flexibly capture potential non-linear relationships.

**Figure 3:**
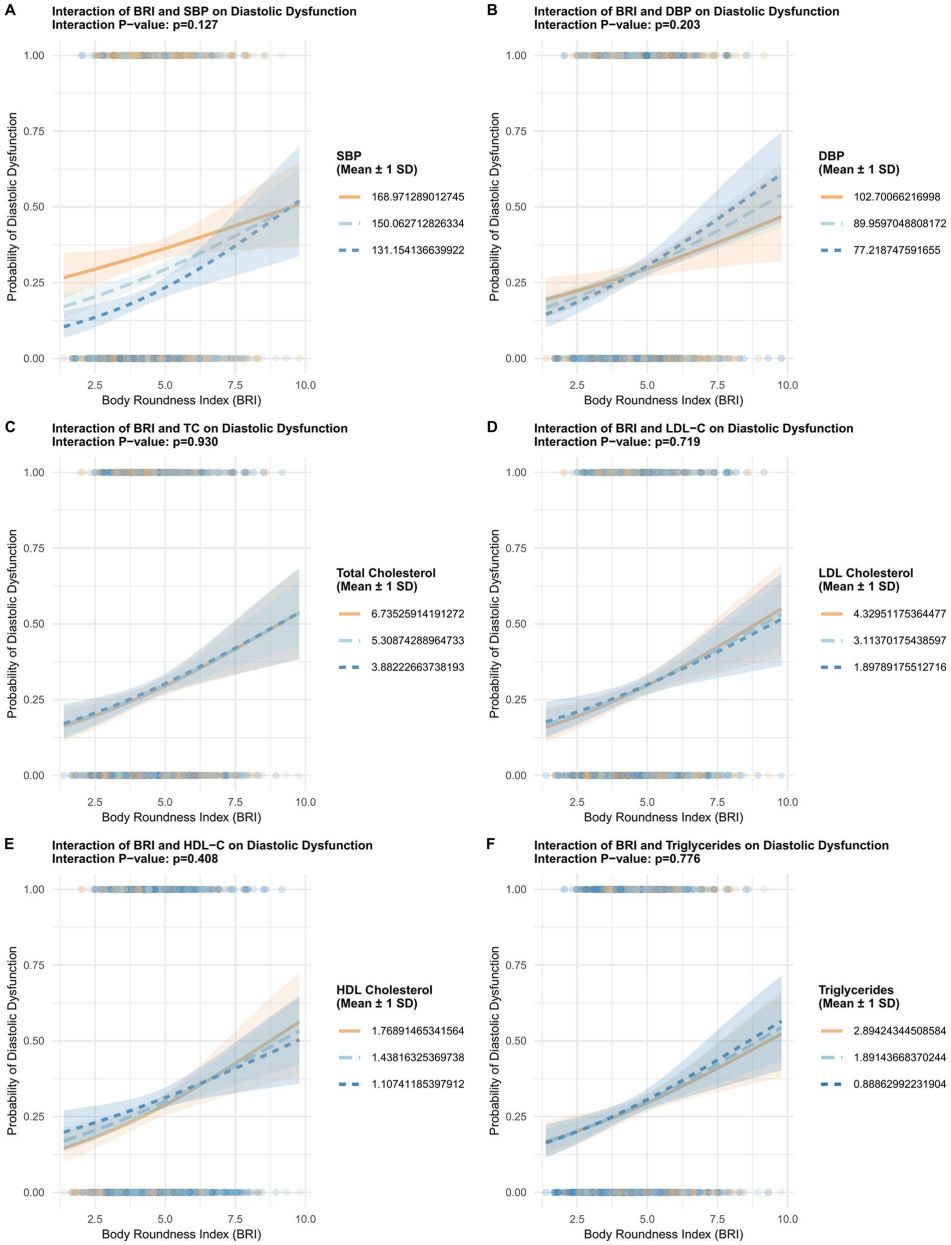
Interaction analyses investigating the modifying effects of blood pressure and lipid profiles on the association between BRI and DD. Panel **A** shows BRI and systolic blood pressure (SBP) with *p* = 0.127. Panel **B** displays BRI and diastolic blood pressure (DBP) with *p* = 0.203. Panel **C** depicts BRI and total cholesterol with *p* = 0.930. Panel **D** presents BRI and LDL cholesterol with *p* = 0.719. Panel **E** illustrates BRI and HDL cholesterol with *p* = 0.408. Panel **F** shows BRI and triglycerides with *p* = 0.776. Each graph features shaded areas representing mean plus or minus one standard deviation.

Restricted cubic spline modeling (Figure 2) showed a nonlinear relationship between BRI and DD probability, with *P* < 0.001 for overall association and *P* = 0.006 for linearity. Interaction analyses (Figure 3) revealed that BRI's effect on DD was not modified by blood pressure and lipid profiles.

### Subgroup analyses

**Figure 4:**
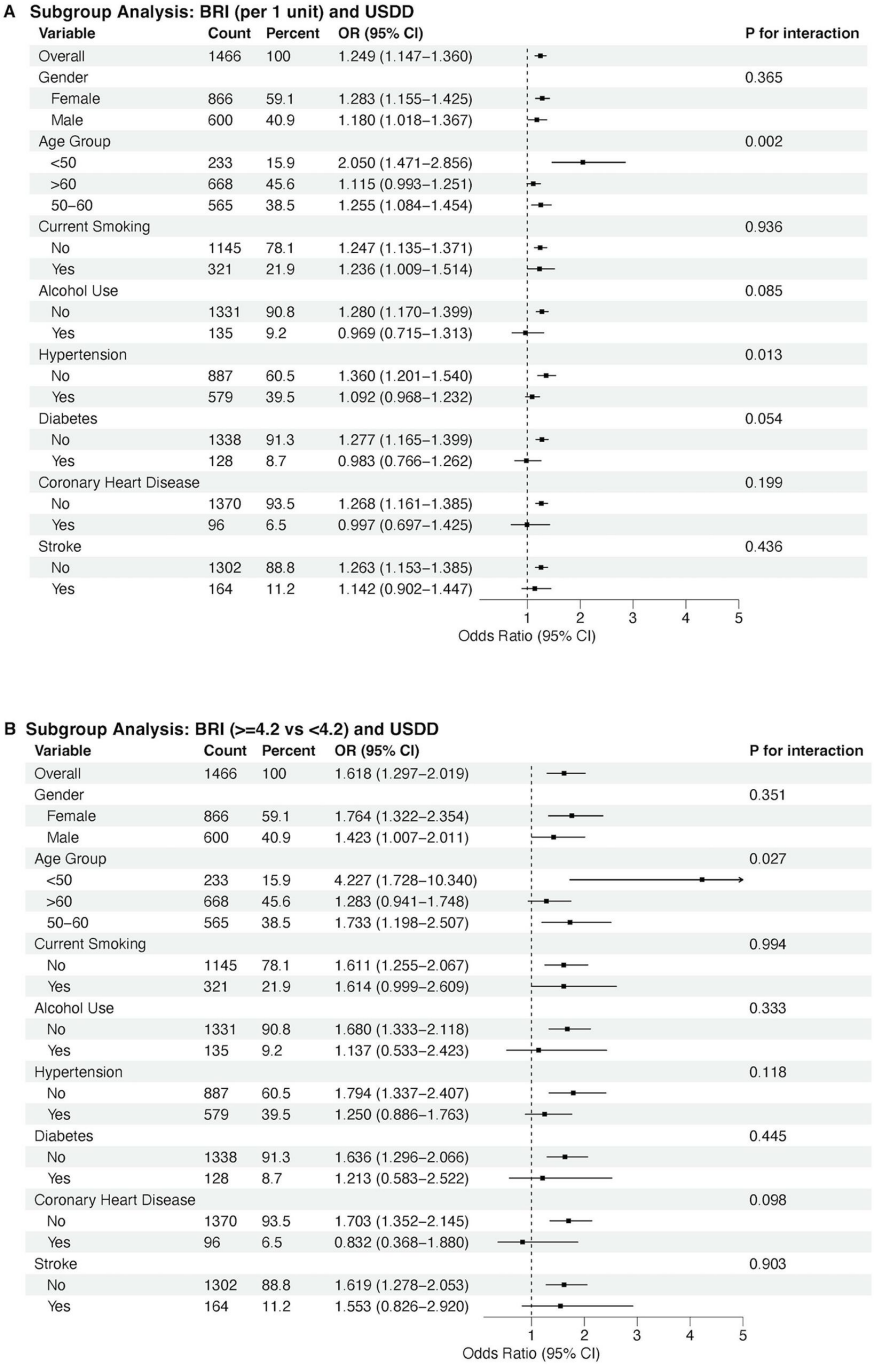
Subgroup analyses by age, sex, current smoking, alcohol use, hypertension, diabetes, coronary heart disease, and stroke. Panel **A** shows associations per 1-unit increase in BRI; Panel **B** compares low BRI (<4.2) and high BRI (≥4.2) groups.

Subgroup analyses by age, sex, current smoking, alcohol use, hypertension, diabetes, coronary heart disease, and stroke indicated a consistent trend of association between BRI and DD in almost all subgroups, either by each 1-unit BRI increase of by high and low levels of BRI (Figure 4).

## Discussion

### Main findings

This study demonstrates a significant association between BRI and DD in middle-aged and older adults, independent of traditional cardiovascular risk factors. Key findings include: 1) BRI exhibited a nonlinear association with DD probability (restricted cubic spline: *P* < 0.001 for overall association, *P* = 0.006 for linearity) and a modest discriminative ability for DD (AUC = 0.67), with an optimal cutoff of 4.2; 2) multivariable regression confirmed BRI's independent association with DD, even after adjusting for traditional cardiovascular risk factors. Each 1-unit BRI increase was associated with 14.4% higher odds of DD (*P* = 0.003) and high BRI (≥4.2) was associated with 41.7% higher odds of DD (*P* = 0.004), and each 1-unit BRI increase correlated with unfavorable changes in continuous diastolic parameters: reduced septal e’ (*β* = −0.164, *P* = 8.10 × 10^−4^) and lateral e’ velocities (*β* = −0.167, *P* = 5.59 × 10^−3^), and a marginally elevated E/e’ ratio (*β* = 0.121, *P* = 0.053). These findings link BRI-an index of body shape and central adiposity-to impaired myocardial relaxation and subclinical filling pressure elevation. These results highlight BRI as a novel adiposity marker for early DD detection.

### Comparison with existing literature

Previous studies have predominantly focused on the association between obesity and cardiac structure, while research on the correlation between obesity and diastolic function remains scarce. This may be attributed to the fact that the assessment of diastolic function imposes relatively high requirements on time and equipment-early cardiac ultrasound examinations typically did not include evaluations of diastolic function, leading to a relative paucity of clinical epidemiological studies incorporating diastolic function indices. Our findings build on and extend this limited evidence: rather than confirming a linear link between adiposity and diastolic dysfunction (DD), we identified a nonlinear association between BRI and DD probability (restricted cubic spline: *P* < 0.001 for overall association, *P* = 0.006 for linearity). This overall association aligns with prior research highlighting central adiposity as a key driver of cardiac remodeling (19–24).

This nonlinearity manifests as a “decelerating risk trajectory”: as BRI increases, the probability of DD rises rapidly in the lower-to-moderate BRI range, but this upward trend slows markedly in the moderate-to-high BRI range. This contrasts with BMI, which often exhibits a J-shaped association with cardiac outcomes (25, 26). The interaction effects observed with blood pressure and lipids further underscore the complex interplay between body shape, hemodynamics, and metabolism in driving DD. For example, BRI's effect on DD risk is more pronounced in participants with lower diastolic blood pressure (DBP), which suggests that in the absence of elevated DBP-a known driver of myocardial stiffening, BRI-related central adiposity becomes a more dominant contributor to DD risk. A similar pattern was observed with triglycerides: lower triglycerides amplified the BRI-DD association, while higher HDL-C mitigated this effect. Collectively, these interactions highlight that BRI is a more impactful marker of DD risk in individuals with less pre-existing cardiometabolic stress (lower DBP, higher HDL-C and lower triglycerides)-reinforcing the value of BRI for early screening in these subgroups, where interventions to reduce central adiposity may most effectively prevent DD progression.

### Clinical implications

BRI offers several advantages over traditional adiposity measures in primary care: 1) It is easily calculable from waist circumference and height, requiring no additional equipment; 2) It captures regional fat distribution, which is more strongly linked to cardiometabolic risk than BMI; and 3) Its linear association with DD allows for graduated risk stratification. Given its modest discriminatory performance (AUC ≈ 0.67), BRI should not be used as a standalone screening tool; however, it may serve as an adjunctive or risk enrichment marker to help prioritize individuals-particularly those with borderline risk profiles-for further echocardiographic evaluation when combined with clinical judgment and other risk factors.

The observed sex and age differences have important screening implications. Younger males with high BRI showed particularly strong associations with DD, highlighting this subgroup as a priority for early intervention. Lifestyle modifications (e.g., diet, exercise) to reduce central adiposity might mitigate DD progression, as supported by prior trials showing that weight loss improves diastolic function.

### Limitations

Several limitations should be considered. First, the cross-sectional design precludes causal inference, and longitudinal studies are needed to establish temporality. Second, BRI was derived from waist circumference and height, potentially missing contributions from visceral fat or muscle mass. Future studies could incorporate dual-energy x-ray absorptiometry or MRI to validate BRI's relationship with body composition. Third, the study population was predominantly Chinese, limiting generalizability to other ethnic groups. Fourth, while bootstrap validation reduced optimism bias in ROC analysis and BRI showed slightly higher AUC than BMI, waist circumference, or BFR, the absolute differences were small and all indices exhibited limited discriminatory accuracy (AUCs < 0.58 in unadjusted models), reinforcing that BRI should be interpreted as a supplementary risk indicator rather than a diagnostic test. Finally, DD was defined using combined echocardiographic parameters, but regional variations in diagnostic criteria may affect reproducibility.

### Future directions

Prospective studies should evaluate whether BRI changes over time predict DD incidence and cardiovascular outcomes. Mechanistic research is needed to clarify how BRI influences myocardial structure and function, including inflammatory and neurohumoral pathways. Additionally, evaluating BRI's utility in conjunction with other markers (e.g., natriuretic peptides) could enhance risk prediction. Finally, randomized controlled trials are needed to test whether BRI-targeted interventions improve diastolic function.

## Conclusion

This study identifies BRI as an independent correlate of diastolic dysfunction in middle-aged and older adults. Given its modest discriminatory performance, BRI is not recommended as a standalone screening tool; however, it may serve as a supplementary anthropometric indicator that reflects body shape related cardiometabolic risk. When considered alongside established clinical and echocardiographic data, BRI could contribute to more nuanced risk stratification in preventive cardiovascular assessment. Future research should focus on validating its incremental value in prospective cohorts and exploring its mechanistic links to myocardial structure and function.

## Data Availability

The raw data supporting the conclusions of this article will be made available by the authors, without undue reservation.

## Supplementary material

Supplementary Figure S1 ROC curves comparing the discriminative ability of waist circumference, BMI, BRI, and BFR for predicting DD.
