# Supplementary figures and images for: Association between Body Roundness Index and diastolic function in middle-aged and older adults: a cross-sectional study
Source: Front Cardiovasc Med. 2026 Jan 12;12:1659587. doi: 10.3389/fcvm.2025.1659587 (PMC12833456; doi:10.3389/fcvm.2025.1659587)

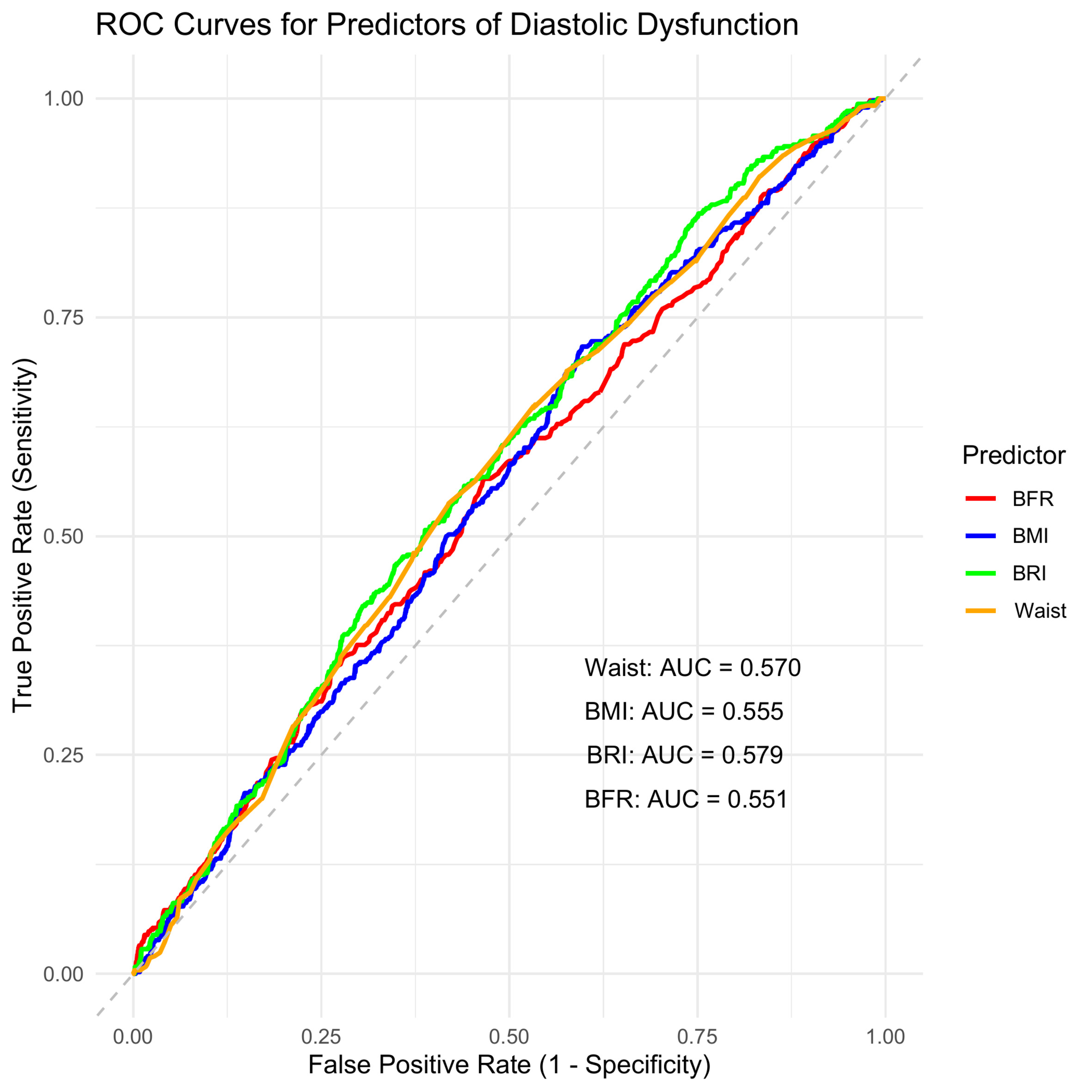

Supplement: Supplementary Figure S1 — ROC curves comparing the discriminative ability of waist circumference, BMI, BRI, and BFR for predicting DD. [file Image1.jpeg]
